# The diagnosis and treatment of venous thromboembolism in asian patients

**DOI:** 10.1186/s12959-017-0155-z

**Published:** 2018-01-18

**Authors:** Kang-Ling Wang, Eng Soo Yap, Shinya Goto, Shu Zhang, Chung-Wah Siu, Chern-En Chiang

**Affiliations:** 1 General Clinical Research Center, Taipei Veterans General Hospital, No. 201, Sec. 2, Shipai Rd., 11217 Taipei, Taiwan; 2 School of Medicine, National Yang-Ming University, Taipei, Taiwan; 3 Department of Haematology-Oncology, National University Cancer Institute, Singapore, Singapore; 4 Department of Laboratory Medicine, National University Hospital, Singapore, Singapore; 5 Department of Medicine, Tokai University School of Medicine, Kanagawa, Japan; 6 Arrhythmia Center, National Center for Cardiovascular Diseases and Beijing Fuwai Hospital, Chinese Academy of Medical Sciences and Pekin Union Medical College, Beijing, China; 7 Cardiology Division, Department of Medicine, Li Ka Shing Faculty of Medicine, The University of Hong Kong, Hong Kong SAR, China

**Keywords:** Venous thromboembolism, Asia, Epidemiology, Risk factors, Treatment

## Abstract

Although the incidence of venous thromboembolism (VTE) in Asian populations is lower than in Western countries, the overall burden of VTE in Asia has been considerably underestimated. Factors that may explain the lower prevalence of VTE in Asian populations relative to Western populations include the limited availability of epidemiological data in Asia, ethnic differences in the genetic predisposition to VTE, underdiagnoses, low awareness toward thrombotic disease, and possibly less symptomatic VTE in Asian patients. The clinical assessment, diagnostic testing, and therapeutic considerations for VTE are, in general, the same in Asian populations as they are in Western populations. The management of VTE is based upon balancing the treatment benefits against the risk of bleeding. This is an especially important consideration for Asian populations because of increased risk of intracranial hemorrhage with vitamin K antagonists. Non-vitamin K antagonist oral anticoagulants have shown advantages over current treatment modalities with respect to bleeding outcomes in major phase 3 clinical trials, including in Asian populations. Although anticoagulant therapy has been shown to reduce the risk of postoperative VTE in Western populations, VTE prophylaxis is not administered routinely in Asian countries. Despite advances in the management of VTE, data in Asian populations on the incidence, prevalence, recurrence, risk factors, and management of bleeding complications are limited and there is need for increased awareness. To that end, this review summarizes the available data on the epidemiology, risk stratification, diagnosis, and treatment considerations in the management of VTE in Asia.

## Background

Venous thromboembolism (VTE), which includes deep vein thrombosis (DVT) and pulmonary embolism (PE), is a significant healthcare burden that remains under-recognized [1–3]. Even with anticoagulant therapy, the mortality rate and the risk of recurrence are high in the early phase of VTE [4–6], and it has serious long-term complications, including chronic pulmonary hypertension and post-thrombotic syndrome, both of which require substantial healthcare resources for their management and are associated with considerable morbidity [7, 8].

VTE is a common cause of preventable mortality for both medical and surgical patients. In addition to early mortality related to PE, VTE associated with hospitalization is a leading cause of lost disability-adjusted life years across low-, middle-, and high-income countries. Although anticoagulant therapy has been shown to reduce the risk of postoperative VTE in Western populations, VTE prophylaxis is not administered routinely in Asian countries [9–11].

The disease burden associated with VTE is high, as the incidence of VTE in Western countries is approximately 100 cases per 100,000 patient-years [12]. The incidence of VTE has risen in Asia over recent years but remains lower than in Western countries [13, 14]. In this review, we summarize the epidemiology, risk stratification, diagnosis, and treatment considerations in the management of VTE in Asia.

### Epidemiology

Although Asian populations are subject to the same major acquired risk factors for VTE as Western populations, studies conducted in Asia have consistently reported lower rates of VTE in Asian populations than in Caucasians (Table 1) [3, 13, 15–20]. These data are comparable to those obtained from Asian patients in Western countries [21, 22]. There are several possibilities that may explain the lower rate of VTE in Asian populations relative to Western populations. Firstly, the estimates may be lower than the true numbers because of the limited availability of epidemiological data in Asia and the asymptomatic nature of VTE. Secondly, historically, the difference in incidence rates reflects underdiagnosis in Asian patients as a result of low awareness toward thrombotic disease and/or manifestations, low clinical suspicion due to the perceived low incidence rate, and limited access to healthcare resources [23–25]. In addition, low autopsy rates—mainly because of cultural and religious practices—may partially account for the perceived low incidence rate of VTE in Asia [25]. Autopsies reveal high rates of asymptomatic thrombosis [2], and autopsy studies indicate that the incidence of PE in Asian countries is comparable with that in Western countries [2, 26, 27]. Finally, the low rates of VTE in Asian populations may be attributed to the low prevalence of risk factors, such as obesity and mutations, in prothrombin or factor V Leiden genes [28–31]. Accordingly, these data suggest that the rate of VTE in Asia may be underestimated, particularly because the thrombi tend not to advance to symptomatic thrombosis in Asian patients [32].

**Table 1:** Estimated incidence of VTE from studies in Western and Asian populations [3, 13, 15–20]

	Western countries			Asian countries				
Incidence^a^	UK	Norway	US (age-adjusted)	Taiwan^b^	Hong Kong	Japan^c^	Korea^c^ (age-adjusted)	Singapore^d^
VTE	75	143	117	16	17	NR	14	57
DVT	40	93	48	NR	NR	12	5	NR
PE	34	50	69	NR	NR	6	7	15

^a^First incidence per 100,000 person-years unless indicated otherwise

^b^Crude incidence

^c^Overall incidence

^d^Overall incidence (Chinese, Indian, Malay)

*DVT* deep vein thrombosis, *NR* not reported, *PE* pulmonary embolism, *VTE* venous thromboembolism

### Risk factors

Heritable risk factors arise from genetic abnormalities in the components of the coagulation pathway that lead to hereditary thrombophilia, including mutations in factor V and prothrombin; and deficiencies of protein S, protein C, and antithrombin [28]. While factor V Leiden and prothrombin G20210A polymorphisms are exclusive to Caucasians, the prevalence of protein S, protein C, and antithrombin deficiencies in Asian populations are higher than those found in Caucasians (Table 2) [30, 33–38].

**Table 2:** Ethnic differences in the distribution of inherited thrombophilias

	Healthy subjects		Patients with VTE	
	Western [123]	Asian [33, 39]	Western [123]	Asian [39]
Factor V Leiden mutation	4.8%	0%–0.2%	18.8%	0%
Prothrombin G20210A mutation	2.7%	0%–0.2%	7.1%	0%
Protein S deficiency	0.03%–0.13%	0.06%–6.4%	2.3%	10.7%–17.8%
Protein C deficiency	0.2%–0.4%	0.3%–4.0%	3.7%	8.9%–10.7%
Antithrombin deficiency	0.02%	0%–6.4%	1.9%	4.7%–8.1%

*VTE* venous thromboembolism

Although the major inherited risk factors for VTE are different between Asian and Western populations, the major acquired risk factors in Asians are similar to those of the Western populations [39]. Risk factors, such as surgery, trauma, prolonged bed rest, immobility, and pregnancy, are transient and reversible, while risk factors, such as malignancy and paralysis due to nerve damages, are irreversible. The most common acquired risk factor for VTE in Asians is malignancy; 16% to 40% of VTE cases are cancer-associated [40–42]. Other common acquired risk factors for VTE in Asians include surgery, immobility, obesity, advanced age, and the use of oral contraceptives [39, 43].

VTE is a serious complication after high-risk surgeries even when preventive measures are taken. The rates for symptomatic DVT and PE with low-molecular-weight heparin (LMWH) after orthopedic surgery are 0.8% and 0.35%, respectively [10]. Since Asian patients have a perceived lower risk for symptomatic VTE following surgery than in Western populations, regular prophylaxis in Asian patients at high risk for VTE is not always administered [44]. However, in studies involving Asian patients undergoing major surgery, the incidence of postoperative DVT was noted to be similar to that reported in Western populations [39, 45–50]. The Assessment of the Incidence of Deep Vein Thrombosis in Asia (AIDA) study, which was conducted in 19 centers across Asia (China, Indonesia, Korea, Malaysia, the Philippines, Taiwan, and Thailand) in patients undergoing total hip or knee arthroplasty or hip fracture surgery and did not receive thromboprophylaxis, assessed the rate of DVT of the lower limbs using bilateral venography; DVT was diagnosed in 41% of patients (121/295) [51]. A meta-analysis of 22 studies done in Asian patients undergoing orthopedic procedures showed that Asian patients have similar overall DVT rates detected by venography, but a lower rate of symptomatic and proximal DVT than Western populations [52]. The Epidemiologic International Day for the Evaluation of Patients at Risk for Venous Thromboembolism in the Acute Hospital Care Setting ENDORSE) study was a multinational cross-sectional survey designed to assess the prevalence of VTE in accordance with the 2004 American College of Chest Physicians (ACCP) guidelines in the acute hospital care setting. In Asian countries (India, Thailand, Pakistan, and Bangladesh), the proportion of surgical patients at risk for VTE ranged from 44% to 62%, which was similar to the proportion reported for all countries studied (overall: 64%; range: 44%–80%) [9]. These findings suggest that surgical patients at risk for VTE in Asian countries should receive appropriate VTE prophylaxis.

### Diagnosis considerations

In general, the clinical assessment and diagnostic testing for VTE are the same in Asian populations as they are in non-Asian populations. DVT usually originates in the deep veins of the calf and can extend into the popliteal and femoral veins [53]. DVT at the calf is generally asymptomatic, but it may produce symptoms once it extends proximally and obstructs venous outflow [53, 54]. Symptomatic DVT is suspected primarily on the basis of unilateral leg pain, swelling, and/or redness [55]. Once extended proximally, venous thrombi may give rise to fatal PE [54]. Common symptoms of PE include palpitation, dyspnea, chest pain, cough, and/or syncope [56, 57].

**Fig. 1:**
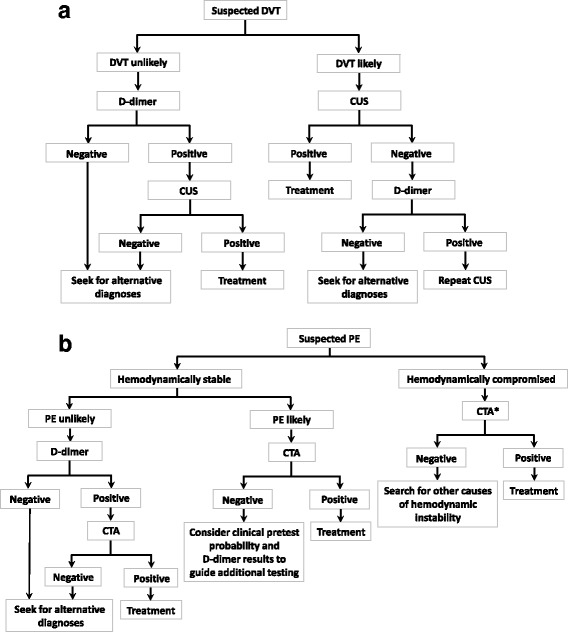
Diagnosis of patients with **a**) suspected DVT; **b**) suspected PE. *When CTA is not available immediately, transthoracic or transesophageal echocardiography indicating mobile right heart thrombi or transesophageal echocardiography indicating main pulmonary arterial thrombi could be otherwise diagnostic. CTA, computed tomographic angiography; CUS, compression ultrasound; DVT, deep vein thrombosis; PE, pulmonary embolism

Careful clinical examinations of signs, symptoms, and risk factors associated with suspected VTE, and distinguishing it from other medical conditions, are important for accurate diagnosis of the disease. Clinical assessment, plasma D-dimer measurement, and imaging tests are recommended and validated for the diagnosis of DVT and PE. The most commonly proposed systematic diagnostic management techniques for VTE are illustrated in Fig. 1.

#### Clinical assessment

The Wells scoring system is the most widely used pretest probability scoring system stratifying patients with suspected DVT or PE [58, 59]. The clinical features used for DVT stratification are (1) active cancer; (2) immobilization of the lower extremities; (3) bed rest for more than 3 days or major surgery within 12 weeks; (4) tenderness along the distribution of the deep venous system; (5) swollen leg; (6) affected calf swelling by more than 3 cm as being compared with the asymptomatic leg; (7) pitting edema; (8) collateral superficial (nonvaricose) veins; and (9) alternative diagnoses as likely as DVT [60]. The clinical features used for PE stratification are (1) signs and symptoms of DVT; (2) heart rate higher than 100 beats/min; (3) immobilization for ≥3 consecutive days or surgery in the previous 4 weeks; (4) previous objectively diagnosed DVT or PE; (5) hemoptysis; (6) active cancer; (7) and PE as likely as, or more likely than, an alternative diagnosis [61]. Although the reliability of the Wells scoring system has been established in Western populations, among Asian countries it has only been validated for DVT in Japan and Singapore with relatively small number of patients [62–64]. Based on the results of 2 Japanese studies, the combination of Wells scoring system and D-dimer testing was effective in excluding DVT and reducing the need for venous duplex scanning. In a study conducted in Singapore, the combination of Wells scoring system and D-dimer testing was effective in reducing unnecessary ultrasound scans for excluding DVT in patients with suspected DVT presenting to the emergency department. Despite these promising results from Asian patients, confirmatory studies with a larger number of patients will help establish the effectiveness of the Wells scoring system in Asia.

#### Diagnostic tests

D-dimer, a degradation product of cross-linked fibrin, is typically elevated in VTE, but also in conditions such as infection, malignancy, pregnancy, surgery, trauma, and stroke [65]. The value of D-dimer testing, due to its moderate specificity, lies in its ability as a negative predictor in patients with suspected DVT or PE when used in combination with clinical pretest probability in both Asian and Western populations [60, 66–70], simplifying the diagnostic process (illustrated in Fig. 1). Although D-dimer testing alone was not accurate enough to detect DVT after total knee arthroplasty in Asian patients [70], it was useful in excluding DVT in hospitalized Japanese patients with acute medical diseases. Among 42 hospitalized patients with acute medical diseases in which plasma D-dimer was measured, the sensitivity and negative predictive value of D-dimer reached 100%, while the positive predictive value (31.6%) and specificity (13.3%) were low [68].

Commercially available D-dimer assays include latex agglutination, whole blood agglutination, and enzyme-linked immunosorbent assays [71]. The Taiwan Society of Cardiology guidelines recommend using D-dimer enzyme-linked immunofluorescence, enzyme-linked immunosorbent, and latex quantitative assays over whole blood, latex semiquantitative, and latex qualitative assays due to their higher sensitivity. Furthermore, since the specificity of D-dimer assay seems to decrease with age, age-adjusted cutoffs (age × 10 μg/L above 50 years) are suggested to improve the specificity of D-dimer testing [72]. Due to the difficulty in standardization of the different available assays, the D-dimer assays used in the diagnostic processes should be of equivalent sensitivity and specificity to ones used in clinical trials in order to be able to compare results obtained with different methods.

In both Asian and Western populations, compression ultrasound (CUS) and multidetector computed tomographic angiography (CTA) have become the methods of choice for effectively imaging the vasculature with high sensitivity and specificity in patients with suspected DVT and PE, respectively [73, 74]. The sensitivity and specificity of CUS for DVT (proximal and distal) is 90.3% and 97.8%, respectively. The sensitivity and specificity of CTA for PE is 83.0% and 96.0%, respectively [75, 76].

### Risk stratification

Selection of patients who are at low or high risk of VTE is crucial when considering prevention options for VTE. The development of VTE in patients is affected by the aforementioned risk factors, such as age, previous history of VTE, cancer, and surgery. Although orthopedic surgeries, such as total hip or knee arthroplasty and hip fracture surgery, are classified as high risk for VTE in Asia, VTE risk stratification is not routine and there is a strong need for a hospital VTE management protocol for VTE risk assessment [77].

The Pulmonary Embolism Severity Index (PESI) and its simplified version (sPESI) are the most extensively used prediction scores for the risk stratification in patients diagnosed with PE in order to guide therapeutic decision making [78, 79]. PE patients with a sPESI score of 0 have a 30-day all-cause mortality rate of 1%, whereas PE patients with a sPESI score of 1 or more have a 30-day all-cause mortality rate of 9% to 11% [79]. PE patients with a sPESI score of 0 are considered to be low risk and may be considered for outpatient treatment [57]. However, patients estimated to be high risk may benefit from inpatient management and/or higher levels of care (ie, intensive care setting). The Hestia criteria are also widely used for selecting patients with PE, including those with right ventricular (RV) dysfunction, for outpatient treatment [80, 81].

The bleeding risk assessment tools may be useful for distinguishing which patients are at low or high risk of bleeding and for identifying patients who might benefit from extended anticoagulation [82–86]. A risk score based on VTE patients included in the RIETE registry identified VTE patients at low, intermediate, or high risk of major bleeding during the first 3 months of therapy. This score was based on 6 variables documented at entry—recent major bleeding, anemia, cancer, abnormal creatinine levels, age > 75 years, and PE diagnosis at baseline [84]. Similarly, the VTE-BLEED score was based on 6 variables—active cancer, male with uncontrolled arterial hypertension, anemia, history of bleeding, age ≥ 60 years, and renal dysfunction—and accurately predicted major bleeding events in VTE patients on anticoagulation [86, 87]. The predictive value of these bleeding risk scores is uncertain in Asian populations and needs to be validated with careful examination of independent risk factors in Asians.

### VTE prevention and treatment guidelines in Asia

The effectiveness of postoperative thromboprophylaxis in Western countries is well recognized [10, 11]. Recent studies conducted in Asia reported that postoperative thromboprophylaxis reduces VTE risk without significantly increasing the risk of bleeding [88]. For the prevention of VTE in patients undergoing high-risk surgeries, thromboprophylaxis with anticoagulants and/or mechanical prophylaxis are typically recommended based on patients’ risk of bleeding [11, 14]. The Asian Venous Thrombosis Forum—composed of experts from China, Hong Kong, Malaysia, the Philippines, Singapore, Taiwan, Thailand, India, Indonesia, Korea, Australia, and Europe—recommends using mechanical prophylaxis for patients with increased risk of bleeding, and mechanical prophylaxis in combination with pharmacological prophylaxis for patients with high risk of VTE [77]. Korean Society of Thrombosis and Hemostasis guidelines also recommend using mechanical prophylaxis for patients with increased risk of bleeding and pharmacological prophylaxis, including LMWH, fondaparinux, dabigatran, apixaban, rivaroxaban, low-dose unfractionated heparin, vitamin K antagonist (VKA; ie, warfarin), or aspirin, for patients undergoing major orthopedic surgery of the lower limbs, such as total hip or knee arthroplasty [89]. According to the latest update, the Asia-Pacific Thrombosis Advisory Board suggests routine use of postoperative thromboprophylaxis for VTE after major orthopedic surgery. They further suggest that the use of non-vitamin K antagonist oral anticoagulants (NOACs) may simplify patient management in Asia primarily due to no regular coagulation monitoring requirement because of their predictable pharmacokinetic (PK) and pharmacodynamic (PD) properties, and demonstrating no interactions with nonsteroidal anti-inflammatory drugs [88]. The Asian Venous Thrombosis Forum recommends LMWH (ie, enoxaparin), fondaparinux, NOACs, VKA, or aspirin with intermittent pneumatic compression for thromboprophylaxis in patients undergoing total hip or knee arthroplasty or hip fracture surgery [77]. Enoxaparin and fondaparinux are the standard therapy for the prevention of VTE in Japanese patients undergoing abdominal surgery or orthopedic surgery of the lower limbs [14]. However, results of a small sized randomized controlled trial conducted in Japan suggested that dabigatran reduces incidence of VTE in patients undergoing total knee arthroplasty with a safety profile comparable to placebo [90]. Furthermore, results from small sized phase 3 trials (STARS [Studying Thrombosis After Replacement Surgery]) indicated that edoxaban is superior to enoxaparin in preventing VTE in Japanese patients undergoing total hip and knee arthroplasty, and has similar safety and efficacy as enoxaparin in hip fracture surgery [91–93]. The results of these clinical trials led to the approval of edoxaban for venous thromboprophylaxis in patients undergoing major orthopedic surgery in Japan [94]. In a postmarketing surveillance study done to monitor the adverse drug reactions of edoxaban during the first 6 months after its commercial launch in Japan, edoxaban’s safety data were consistent with its known safety profile [95].

**Fig. 2:**
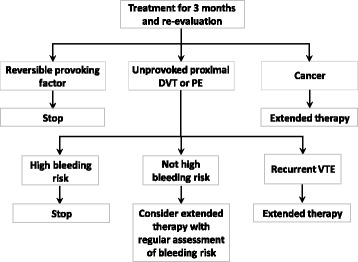
Risk-benefit analysis of extended therapy for VTE. DVT, deep vein thrombosis; PE, pulmonary embolism; VTE, venous thromboembolism

The goal of the VTE treatment is to prevent thrombus extension and recurrence through pharmacological or mechanical interventions [96]. Japanese and Taiwanese guidelines were issued by the Japanese Circulation Society (JCS) in 2011 and by the Taiwan Society of Cardiology in 2016 [14, 72]. The ACCP VTE treatment guidelines and European Society of Cardiology PE guidelines are also widely used in Asia [57, 97]. According to the latest update, treatment with NOACs is suggested over VKA therapy. The suggested duration of treatment for symptomatic DVT (distal or proximal) or PE is at least 3 months, and patients should be evaluated for the risk-benefit ratio to determine the need for extended therapy (no scheduled stop date) (Fig. 2) [96]. However, the JCS guidelines recommend using intravenous unfractionated heparin (UFH) overlapped with, and followed by, VKA for a minimum of 3 months, and the recommended target international normalized ratio (INR) range is 1.5 – 2.5. This target INR range is lower than the range recommended in Western countries (ie, 2.0–3.0) perhaps because of increased bleeding tendency in Japanese patients [14, 43]. Although use of LMWH was adopted in the US and Europe to overcome the limitations of UFH for the treatment of VTE, LMWH has yet to be approved for this indication in Japan due to limited clinical evidence from Japanese patients [98]. The Taiwanese guidelines recommend using either intravenous UFH or LMWH overlapped with, and followed by, VKA with a maintenance target INR of 2.0 to 3.0 [72].

NOACs have been approved for the treatment of VTE in many countries in Asia; however, only a few countries provide reimbursements to patients. Dabigatran has been approved in Korea, Singapore, the Philippines, China, Thailand, and Taiwan; rivaroxaban and apixaban have been approved in Korea, Singapore, Japan, China, Thailand, and Taiwan; and edoxaban has been approved in Japan, South Korea, Hong Kong, Thailand, and Taiwan at the time of this review [94, 99, 100].

### Anticoagulants for the treatment of VTE

#### Parenteral anticoagulants

Treatment of DVT and PE has traditionally been initial parenteral anticoagulation overlapping with, or followed by, longer-term VKAs [96]. While the UFH therapy has been proven effective in anticoagulation, it has limitations that include the requirement for activated partial thromboplastin time monitoring and the risk of heparin-induced thrombocytopenia and osteoporosis. On the contrary, LMWH and fondaparinux have predictable PK and PD properties and are associated with a lower risk of nonhemorrhagic side effects [101]. On average, Asian patients have lower body mass index than non-Asian populations [102]. Weight-based dose adjustments without routine monitoring are required for parenteral anticoagulants [101, 103]; however, the need for parenteral administration limits their use for outpatient treatment [104]. Regular monitoring of LMWH therapy is recommended only for patients who have an increased risk of bleeding, such as patients with extremely low or high body weight, renal insufficiency (creatinine clearance <30 mL/min), and advanced age [105–107].

#### Vitamin K antagonists

VKAs have been the most widely used anticoagulants for the treatment of VTE, but they have several limitations in terms of patient acceptance, including a slow onset and offset of action and a narrow therapeutic window that requires individualized dosing based on INR with the need for regular monitoring [108]. In addition, Asian patients are at an increased risk for bleeding when treated with VKAs. As shown in a post hoc analysis of RE-LY (Randomized Evaluation of Long-term Anticoagulation Therapy), ROCKET AF (Rivaroxaban Once Daily Oral Direct Factor Xa Inhibition Compared with Vitamin K Antagonism for Prevention of Stroke and Embolism Trial in Atrial Fibrillation), and ARISTOTLE (Apixaban for Reduction in Stroke and Other Thromboembolic Events in Atrial Fibrillation) trials, VKA use in Asian patients with atrial fibrillation (AF) is associated with a higher risk of bleeding when compared with non-Asian patients [109]. There is limited information on risk of bleeding with VKAs in Asian patients with VTE. However, according to the available reports from the Hokusai-VTE trial, Asian patients with VTE randomized to VKAs had higher rates of overall and major or clinically relevant nonmajor (CRNM) bleeding than non-Asian patients randomized to VKAs [110]. In a subgroup analysis that examined the results of the Chinese patients included in the EINSTEIN-DVT (Oral Direct Factor Xa Inhibitor Rivaroxaban in Patients with Acute Symptomatic Deep Vein Thrombosis) and EINSTEIN-PE (Oral Direct Factor Xa Inhibitor Rivaroxaban in Patients with Acute Symptomatic Pulmonary Embolism) trials, 9.2% (20/218) of patients receiving VKA therapy experienced a major or CRNM bleeding event [111]. In the AMPLIFY-J (Apixaban for the Initial Management of Pulmonary Embolism and Deep Vein Thrombosis as First-Line Therapy-Japan) trial, 28.2% (11/39) of Japanese patients receiving VKA therapy experienced a major or CRNM bleeding event [112]. On the basis of these data, alternatives to VKAs for the treatment of VTE may be of particular importance for Asian populations.

#### Non-vitamin K antagonist oral anticoagulants

The rapid onset of action, minimal drug and food interactions, predictable PKs, no regular monitoring requirement, and lower risk of bleeding make NOACs an attractive alternative to VKAs [108]. These attributes also make NOACs more applicable for outpatient treatment. The NOACs include the direct thrombin inhibitor dabigatran and the direct factor Xa inhibitors rivaroxaban, apixaban, and edoxaban [113]. Table 3 shows a summary of the efficacy and safety outcomes of clinical trials with NOACs for the treatment of VTE, including in Asian patients.

**Table 3:** Efficacy and safety outcomes of clinical trials with NOACs for the treatment of VTE

All Patients				Asian Patients			
Trial	N	VTE recurrence^a^	Major or CRNM bleeding^a^	Trial	N	VTE recurrence^a^	Major or CRNM bleeding^a^
**Dabigatran**				**Dabigatran**			
RE-COVER [114]	2539	1.10 (0.65–1.84)	*0.63 (0.47–0.84)*	RE-COVER RE-COVER II Asian subanalysis^b^	557	2.55 (0.66–9.90)	0.63 (0.33–1.19)
RE-COVER II [115]	2568	1.08 (0.64–1.80)	*0.62 (0.45–0.84)*				
**Rivaroxaban**				**Rivaroxaban**			
EINSTEIN-DVT [116]	3449	0.68 (0.44–1.04)	0.97 (0.76–1.22)	EINSTEIN-DVT and PE Asian subanalysis [111]	439	1.04 (0.36–3.0)	0.63 (0.31–1.26)
EINSTEIN-PE [117]	4832	1.12 (0.75–1.68)	0.90 (0.76–1.07)	J-EINSTEIN DVT and PE [73]	100	3.9% (−3.4 to 23.8)^c^	Rivaroxaban 7.8% UFH/warfarin 5.3%^d^
**Apixaban**				**Apixaban**			
AMPLIFY [119]	5395	0.84 (0.60–1.18)	*0.44 (0.36–0.55)*	AMPLIFY-J [112]	80	Apixaban 0/40 UFH/warfarin 1/40^e^	Apixaban 7.5% UFH/warfarin 28.2%^f^
**Edoxaban**				**Edoxaban**			
Hokusai-VTE [120]	8240	0.89 (0.70–1.13)	*0.81 (0.71–0.94)*	Hokusai-VTE Asian subanalysis [110]	1109	0.64 (0.34–1.19)	*0.56 (0.40–0.78)*

^a^Values are hazard ratio (95% confidence interval) unless otherwise indicated

^b^Data on file

^c^Absolute risk difference (95% confidence interval)

^d^Percentage of patients with CRNM bleeding

^e^Number of patients

^f^Percentage of patients

*CRNM* clinically relevant nonmajor, *DVT* deep vein thrombosis, *NOAC* non-vitamin K antagonist oral anticoagulant, *PE* pulmonary embolism, *UFH* unfractionated heparin, *VTE* venous thromboembolism

In both RE-COVER (Efficacy and Safety of Dabigatran Compared to Warfarin for 6 Month Treatment of Acute Symptomatic Venous Thromboembolism) and RE-COVER II trials, patients were randomized to receive dabigatran (150 mg twice daily) or warfarin for 6 months after initial parenteral anticoagulation therapy. Both studies indicated that dabigatran is as efficacious as warfarin, with respect to recurrent VTE, and has a lower risk of CRNM bleeding. Although only 2.6% (65/2539) of patients with acute VTE enrolled in the RE-COVER trial were Asian, 20.9% (537/2568) of patients in the RE-COVER II trial were Asian [114, 115].

Both EINSTEIN-DVT and EINSTEIN-PE trials compared rivaroxaban (15 mg twice daily for 3 weeks, followed by 20 mg once daily) with subcutaneous enoxaparin followed by VKA therapy for 3, 6, or 12 months. In both trials, rivaroxaban was as efficacious as the conventional therapy with respect to recurrent VTE, with a similar safety profile with respect to major or CRNM bleeding [116, 117]. A subgroup analysis examined the results of the Chinese patients included in the EINSTEIN-DVT and EINSTEIN-PE trials. The relative efficacy and safety of rivaroxaban compared with the conventional therapy in Chinese patients were consistent with that of the overall population [111]. However, the incidence of major or CRNM bleeding for rivaroxaban was lower in Chinese patients compared with the overall population (5.9% vs 9.4%) [111, 118].

J-EINSTEIN DVT and PE trial compared rivaroxaban (10 or 15 mg twice daily for 3 weeks, followed by 15 mg once daily) with UFH/warfarin for 3, 6, or 12 months in Japanese patients. The relative efficacy of rivaroxaban compared with UFH/warfarin in Japanese patients was consistent with that of the overall population of EINSTEIN-DVT and EINSTEIN-PE. Major bleeding did not occur during the study, and CRNM bleeding occurred in 7.8% (6/77) of patients in the rivaroxaban group and 5.3% (1/19) of patients in the UFH/warfarin group [73]. These results suggest the safety of rivaroxaban for the treatment of VTE in Asian patients.

In the AMPLIFY trial, patients with acute VTE were randomly assigned to apixaban (10 mg twice daily for 7 days, followed by 5 mg twice daily) or subcutaneous enoxaparin followed by warfarin for 6 months. Overall, apixaban was found to be noninferior to the conventional therapy, with respect to recurrent VTE, and had a significant lower risk of major or CRNM bleeding [119]. AMPLIFY-J, which was designed based on the AMPLIFY study, compared apixaban with UFH/warfarin for 24 weeks in Japanese patients with acute VTE. Recurrent VTE did not occur in patients receiving apixaban, but occurred in 1 patient receiving UFH/warfarin. Apixaban had a lower risk of major or CRNM bleeding compared with UFH/warfarin, suggesting the safety of apixaban for the treatment of VTE in Japanese patients [112].

Hokusai-VTE trial compared edoxaban (60 mg [reduced to 30 mg in patients with a creatinine clearance 30–50 mL/min or a body weight ≤ 60 kg or in patients receiving potent P-glycoprotein inhibitors concomitantly] once daily) with warfarin for 3 to 12 months after initial heparin therapy. Edoxaban was as efficacious as warfarin, with respect to recurrent VTE, and had a significantly lower risk of major or CRNM bleeding [120]. In a subgroup of PE patients with evident RV dysfunction (N-terminal probrain natriuretic peptide level of ≥500 pg/mL), edoxaban, compared with warfarin, was associated with a lower rate of recurrent VTE. A subgroup analysis evaluated the results of the East Asian patients included in the Hokusai-VTE trial. The relative efficacy of edoxaban compared with warfarin in East Asian patients was consistent with that of the overall population; however, edoxaban had a better safety profile with respect to major or CRNM bleeding than warfarin in East Asian patients, as compared to that of the overall population, confirming the safety of edoxaban for the treatment of VTE in Asia [110]. Hokusai-VTE is the only VTE trial where a NOAC was dose-adjusted by weight or creatinine clearance. Since Asians are known to have lower body mass index than non-Asian populations, future phase 2/3 and PK/PD studies, specifically in Asian patients, will afford new treatment algorithms and dosing regimens by increasing the understanding of specific characteristics of VTE in Asia.

Taken together, based on the results of these pivotal clinical trials, NOACs provide a strong alternative to conventional therapy with similar efficacy and superior safety profiles in the treatment of VTE [121]. Importantly, the results of the analyses that evaluated NOACs in Asian patients and Asian subgroup analyses—specifically EINSTEIN-DVT, EINSTEIN-PE, and Hokusai-VTE—suggest that NOACs present a possible safety advantage for the treatment of VTE in Asian populations. However, the lack of clinical trials assessing the efficacy and safety of NOACs for the treatment and prevention of VTE specifically in Asian populations makes it difficult to change the standard of care in Asian countries.

## Conclusions

The overall burden of VTE in Asia has been considerably underestimated. Despite advances in the management of VTE globally, more data on epidemiology in the form of first incidence, prevalence, recurrence and risk factors, management of bleeding complications, as well as increased awareness in Asian populations, are necessary. Although regional standard of care may vary based upon physicians’ preference or clinical experience, increased collaborative studies among the Asian countries and participation in international trials may lead to different treatment algorithms and dosing regimens by providing more data on the epidemiology, pharmacology, and bleeding complications of the disease in Asian patients which may differ significantly from Western populations. The ongoing international registry of acute VTE, which includes a substantial number of patients from both Asian and Western countries, will provide important insights for understanding specific characteristics of VTE in Asia [122]. VTE requires considerable healthcare resources for its management due to its chronic nature, high recurrence rate, and associated long-term complications. Decisions for VTE management are based upon balancing the treatment benefits against the risk of bleeding from the treatment. This is an especially important consideration for Asian populations because of increased bleeding tendency of Asians, intracranial hemorrhage in particular. Given this risk, timely and accurate diagnosis of the disease and ruling it out safely when absent are crucial. NOACs have shown advantages over existing options with respect to bleeding outcomes in major clinical trials, which renders them a safe and preferable strategy for VTE treatment.

## Abbreviations

ACCP: American College of Chest Physicians
AF: Atrial fibrillation
AIDA: Assessment of the Incidence of Deep Vein Thrombosis in Asia
AMPLIFY: Apixaban for the Initial Management of Pulmonary Embolism and Deep Vein Thrombosis as First-Line Therapy
ARISTOTLE: Apixaban for Reduction in Stroke and Other Thromboembolic Events in Atrial Fibrillation
CRNM: Clinically relevant nonmajor
CTA: Computed tomographic angiography
CUS: Compression ultrasound
DVT: Deep vein thrombosis
EINSTEIN-DVT: Oral Direct Factor Xa Inhibitor Rivaroxaban in Patients with Acute Symptomatic Deep Vein Thrombosis
EINSTEIN-PE: Oral Direct Factor Xa Inhibitor Rivaroxaban in Patients with Acute Symptomatic Pulmonary Embolism
ENDORSE: Epidemiologic International Day for the Evaluation of Patients at Risk for Venous Thromboembolism in the Acute Hospital Care Setting
INR: International normalized ratio
JCS: Japanese Circulation Society
LMWH: Low-molecular-weight heparin
NOAC: Non-vitamin K antagonist oral anticoagulant
PD: Pharmacodynamic
PESI: Pulmonary Embolism Severity Index
PK: Pharmacokinetic
RE-COVER: Efficacy and Safety of Dabigatran Compared to Warfarin for 6 Month Treatment of Acute Symptomatic Venous Thromboembolism
RE-LY: Randomized Evaluation of Long-term Anticoagulation Therapy
ROCKET AF: Rivaroxaban Once Daily Oral Direct Factor Xa Inhibition Compared with Vitamin K Antagonism for Prevention of Stroke and Embolism Trial in Atrial Fibrillation
RV: Right ventricular
SPESI: Simplified Pulmonary Embolism Severity Index
STARS: Studying Thrombosis After Replacement Surgery
UFH: Unfractionated heparin
VKA: Vitamin K antagonist
VTE: Venous thromboembolism

## References

[1] Vaitkus PT, Leizorovicz A, Cohen AT, Turpie AG, Olsson CG, Goldhaber SZ, Group PMTS (2005). Mortality rates and risk factors for asymptomatic deep vein thrombosis in medical patients. Thromb Haemost.

[2] Sandler DA, Martin JF (1989). Autopsy proven pulmonary embolism in hospital patients: are we detecting enough deep vein thrombosis?. J R Soc Med.

[3] Sakuma M, Nakamura M, Yamada N, Ota S, Shirato K, Nakano T, Ito M, Kobayashi T (2009). Venous thromboembolism: deep vein thrombosis with pulmonary embolism, deep vein thrombosis alone, and pulmonary embolism alone. Circ J.

[4] Goldhaber SZ, Visani L, De Rosa M (1999). Acute pulmonary embolism: clinical outcomes in the international cooperative pulmonary embolism registry (ICOPER). Lancet.

[5] Nijkeuter M, Sohne M, Tick LW, Kamphuisen PW, Kramer MH, Laterveer L, van Houten AA, Kruip MJ, Leebeek FW, Buller HR (2007). The natural course of hemodynamically stable pulmonary embolism: clinical outcome and risk factors in a large prospective cohort study. Chest.

[6] Heit JA, Lahr BD, Petterson TM, Bailey KR, Ashrani AA, Melton LJ (2011). Heparin and warfarin anticoagulation intensity as predictors of recurrence after deep vein thrombosis or pulmonary embolism: a population-based cohort study. Blood.

[7] Pengo V, Lensing AW, Prins MH, Marchiori A, Davidson BL, Tiozzo F, Albanese P, Biasiolo A, Pegoraro C, Iliceto S (2004). Incidence of chronic thromboembolic pulmonary hypertension after pulmonary embolism. N Engl J Med.

[8] Cohen AT, Agnelli G, Anderson FA, Arcelus JI, Bergqvist D, Brecht JG, Greer IA, Heit JA, Hutchinson JL, Kakkar AK (2007). Venous thromboembolism (VTE) in Europe. The number of VTE events and associated morbidity and mortality. Thromb Haemost.

[9] Cohen AT, Tapson VF, Bergmann JF, Goldhaber SZ, Kakkar AK, Deslandes B, Huang W, Zayaruzny M, Emery L, Anderson FA, Investigators E (2008). Venous thromboembolism risk and prophylaxis in the acute hospital care setting (ENDORSE study): a multinational cross-sectional study. Lancet.

[10] Falck-Ytter Y, Francis CW, Johanson NA, Curley C, Dahl OE, Schulman S, Ortel TL, Pauker SG, Colwell CW, American College of Chest P (2012). Prevention of VTE in orthopedic surgery patients: antithrombotic therapy and prevention of thrombosis, 9th ed: American College of Chest Physicians Evidence-Based Clinical Practice Guidelines. Chest.

[11] Gould MK, Garcia DA, Wren SM, Karanicolas PJ, Arcelus JI, Heit JA, Samama CM, American College of Chest P (2012). Prevention of VTE in nonorthopedic surgical patients: antithrombotic therapy and prevention of thrombosis, 9th ed: American College of Chest Physicians Evidence-Based Clinical Practice Guidelines. Chest.

[12] White RH (2003). The epidemiology of venous thromboembolism. Circulation.

[13] Jang MJ, Bang SM, Oh D (2011). Incidence of venous thromboembolism in Korea: from the Health Insurance Review and Assessment Service database. J Thromb Haemost.

[14] JCS (2011). Guidelines for the diagnosis, treatment and prevention of pulmonary thromboembolism and deep vein thrombosis (JCS 2009). Circ J.

[15] Huerta C, Johansson S, Wallander MA, Garcia Rodriguez LA (2007). Risk factors and short-term mortality of venous thromboembolism diagnosed in the primary care setting in the United Kingdom. Arch Intern Med.

[16] Naess IA, Christiansen SC, Romundstad P, Cannegieter SC, Rosendaal FR, Hammerstrom J (2007). Incidence and mortality of venous thrombosis: a population-based study. J Thromb Haemost.

[17] Silverstein MD, Heit JA, Mohr DN, Petterson TM, O'Fallon WM, Melton LJ (1998). Trends in the incidence of deep vein thrombosis and pulmonary embolism: a 25-year population-based study. Arch Intern Med.

[18] Lee CH, Lin LJ, Cheng CL, Kao Yang YH, Chen JY, Tsai LM (2010). Incidence and cumulative recurrence rates of venous thromboembolism in the Taiwanese population. J Thromb Haemost.

[19] Liu HS, Kho BC, Chan JC, Cheung FM, Lau KY, Choi FP, Wu WC, Yau TK (2002). Venous thromboembolism in the Chinese population--experience in a regional hospital in Hong Kong. Hong Kong Med J.

[20] Molina JA, Jiang ZG, Heng BH, Ong BK (2009). Venous thromboembolism at the National Healthcare Group, Singapore. Ann Acad Med Singap.

[21] Liao S, Woulfe T, Hyder S, Merriman E, Simpson D, Chunilal S (2014). Incidence of venous thromboembolism in different ethnic groups: a regional direct comparison study. J Thromb Haemost.

[22] White RH, Zhou H, Murin S, Harvey D (2005). Effect of ethnicity and gender on the incidence of venous thromboembolism in a diverse population in California in 1996. Thromb Haemost.

[23] Zakai NA, McClure LA (2011). Racial differences in venous thromboembolism. J Thromb Haemost.

[24] Wendelboe AM, McCumber M, Hylek EM, Buller H, Weitz JI, Raskob G, Day ISCfWT (2015). Global public awareness of venous thromboembolism. J Thromb Haemost.

[25] Lee LH (2002). Clinical update on deep vein thrombosis in Singapore. Ann Acad Med Singap.

[26] Kakkar N, Vasishta RK (2008). Pulmonary embolism in medical patients: an autopsy-based study. Clin Appl Thromb Hemost.

[27] Dickens P, Knight BH, Ip P, Fung WS (1997). Fatal pulmonary embolism: a comparative study of autopsy incidence in Hong Kong and Cardiff, Wales. Forensic Sci Int.

[28] Margaglione M, Grandone E (2011). Population genetics of venous thromboembolism. A narrative review. Thromb Haemost.

[29] Barnes PM, Adams PF, Powell-Griner E: Health characteristics of the Asian adult population: United States, 2004–2006. *Adv Data* 2008:1–22. 18271366

[30] Jun ZJ, Ping T, Lei Y, Li L, Ming SY, Jing W (2006). Prevalence of factor V Leiden and prothrombin G20210A mutations in Chinese patients with deep venous thrombosis and pulmonary embolism. Clin Lab Haematol.

[31] Ageno W, Becattini C, Brighton T, Selby R, Kamphuisen PW (2008). Cardiovascular risk factors and venous thromboembolism: a meta-analysis. Circulation.

[32] Lee WS, Kim KI, Lee HJ, Kyung HS, Seo SS (2013). The incidence of pulmonary embolism and deep vein thrombosis after knee arthroplasty in Asians remains low: a meta-analysis. Clin Orthop Relat Res.

[33] Shen MC, Lin JS, Tsay W (2000). Protein C and protein S deficiencies are the most important risk factors associated with thrombosis in Chinese venous thrombophilic patients in Taiwan. Thromb Res.

[34] Suehisa E, Nomura T, Kawasaki T, Kanakura Y (2001). Frequency of natural coagulation inhibitor (antithrombin III, protein C and protein S) deficiencies in Japanese patients with spontaneous deep vein thrombosis. Blood Coagul Fibrinolysis.

[35] Akkawat B, Rojnuckarin P (2005). Protein S deficiency is common in a healthy Thai population. J Med Assoc Thail.

[36] Rees DC, Cox M, Clegg JB (1995). World distribution of factor V Leiden. Lancet.

[37] Rosendaal FR, Doggen CJ, Zivelin A, Arruda VR, Aiach M, Siscovick DS, Hillarp A, Watzke HH, Bernardi F, Cumming AM (1998). Geographic distribution of the 20210 G to a prothrombin variant. Thromb Haemost.

[38] Ho CH, Chau WK, Hsu HC, Gau JP, Yu TJ (2000). Causes of venous thrombosis in fifty Chinese patients. Am J Hematol.

[39] Angchaisuksiri P (2011). Venous thromboembolism in Asia--an unrecognised and under-treated problem?. Thromb Haemost.

[40] Peng YY, Jeng JS, Shen MC, Tsay W, Wang BS, Lin WH, Chang YC, Yip PK (1998). Aetiologies and prognosis of Chinese patients with deep vein thrombosis of the lower extremities. QJM.

[41] Lee HC, Liao WB, Bullard MJ, Hsu TS (1996). Deep venous thrombosis in Taiwan. Jpn Heart J.

[42] Mutirangura P, Rüengsethakit C, Wongwanit C (2004). Epidemiologic analysis of proximal deep vein thrombosis in Thai patients: malignancy, the predominant etiologic factor. Int J Angiol.

[43] Nakamura M, Miyata T, Ozeki Y, Takayama M, Komori K, Yamada N, Origasa H, Satokawa H, Maeda H, Tanabe N (2014). Current venous thromboembolism management and outcomes in Japan. Circ J.

[44] Chung LH, Chen WM, Chen CF, Chen TH, Liu CL (2011). Deep vein thrombosis after total knee arthroplasty in asian patients without prophylactic anticoagulation. Orthopedics.

[45] Wang CJ, Wang JW, Chen LM, Chen HS, Yang BY, Cheng SM (2000). Deep vein thrombosis after total knee arthroplasty. J Formos Med Assoc.

[46] Dhillon KS, Askander A, Doraismay S (1996). Postoperative deep-vein thrombosis in Asian patients is not a rarity: a prospective study of 88 patients with no prophylaxis. J Bone Joint Surg Br.

[47] Kadono Y, Yasunaga H, Horiguchi H, Hashimoto H, Matsuda S, Tanaka S, Nakamura K (2010). Statistics for orthopedic surgery 2006-2007: data from the Japanese diagnosis procedure combination database. J Orthop Sci.

[48] Fujita Y, Nakatsuka H, Namba Y, Mitani S, Yoshitake N, Sugimoto E, Hazama K (2015). The incidence of pulmonary embolism and deep vein thrombosis and their predictive risk factors after lower extremity arthroplasty: a retrospective analysis based on diagnosis using multidetector CT. J Anesth.

[49] Liew NC, Moissinac K, Gul Y (2003). Postoperative venous thromboembolism in Asia: a critical appraisal of its incidence. Asian J Surg.

[50] Wu PK, Chen CF, Chung LH, Liu CL, Chen WM (2014). Population-based epidemiology of postoperative venous thromboembolism in Taiwanese patients receiving hip or knee arthroplasty without pharmacological thromboprophylaxis. Thromb Res.

[51] Piovella F, Wang CJ, Lu H, Lee K, Lee LH, Lee WC, Turpie AG, Gallus AS, Planes A, Passera R (2005). Deep-vein thrombosis rates after major orthopedic surgery in Asia. An epidemiological study based on postoperative screening with centrally adjudicated bilateral venography. J Thromb Haemost.

[52] Kanchanabat B, Stapanavatr W, Meknavin S, Soorapanth C, Sumanasrethakul C, Kanchanasuttirak P (2011). Systematic review and meta-analysis on the rate of postoperative venous thromboembolism in orthopaedic surgery in Asian patients without thromboprophylaxis. Br J Surg.

[53] Kakkar VV, Howe CT, Flanc C, Clarke MB (1969). Natural history of postoperative deep-vein thrombosis. Lancet.

[54] Hirsh J, Hoak J (1996). Management of deep vein thrombosis and pulmonary embolism. A statement for healthcare professionals. Council on thrombosis (in consultation with the council on cardiovascular radiology), American Heart Association. Circulation.

[55] Wells P, Anderson D (2013). The diagnosis and treatment of venous thromboembolism. Hematology Am Soc Hematol Educ Program.

[56] Prandoni P, Lensing AW, Prins MH, Ciammaichella M, Perlati M, Mumoli N, Bucherini E, Visona A, Bova C, Imberti D (2016). Prevalence of pulmonary embolism among patients hospitalized for syncope. N Engl J Med.

[57] Konstantinides SV, Torbicki A, Agnelli G, Danchin N, Fitzmaurice D, Galie N, Gibbs JS, Huisman MV, Humbert M, Kucher N (2014). 2014 ESC guidelines on the diagnosis and management of acute pulmonary embolism. Eur Heart J.

[58] Wells PS, Hirsh J, Anderson DR, Lensing AW, Foster G, Kearon C, Weitz J, D'Ovidio R, Cogo A, Prandoni P (1995). Accuracy of clinical assessment of deep-vein thrombosis. Lancet.

[59] Wells PS, Ginsberg JS, Anderson DR, Kearon C, Gent M, Turpie AG, Bormanis J, Weitz J, Chamberlain M, Bowie D (1998). Use of a clinical model for safe management of patients with suspected pulmonary embolism. Ann Intern Med.

[60] Wells PS, Anderson DR, Rodger M, Forgie M, Kearon C, Dreyer J, Kovacs G, Mitchell M, Lewandowski B, Kovacs MJ (2003). Evaluation of D-dimer in the diagnosis of suspected deep-vein thrombosis. N Engl J Med.

[61] Wells PS, Anderson DR, Rodger M, Stiell I, Dreyer JF, Barnes D, Forgie M, Kovacs G, Ward J, Kovacs MJ (2001). Excluding pulmonary embolism at the bedside without diagnostic imaging: management of patients with suspected pulmonary embolism presenting to the emergency department by using a simple clinical model and d-dimer. Ann Intern Med.

[62] H'Ng MW, Loh SS, Earnest A, Wansaicheong GK (2012). Effectiveness of an algorithm in reducing the number of unnecessary ultrasound scans for deep vein thrombosis: an evaluation report. Singap Med J.

[63] Yamaki T, Nozaki M, Sakurai H, Takeuchi M, Soejima K, Kono T (2007). Uses of different D-dimer levels can reduce the need for venous duplex scanning to rule out deep vein thrombosis in patients with symptomatic pulmonary embolism. J Vasc Surg.

[64] Yamaki T, Nozaki M, Sakurai H, Kikuchi Y, Soejima K, Kono T, Hamahata A, Kim K (2009). Combined use of pretest clinical probability score and latex agglutination D-dimer testing for excluding acute deep vein thrombosis. J Vasc Surg.

[65] Righini M, Perrier A, De Moerloose P, Bounameaux H (2008). D-Dimer for venous thromboembolism diagnosis: 20 years later. J Thromb Haemost.

[66] van Belle A, Buller HR, Huisman MV, Huisman PM, Kaasjager K, Kamphuisen PW, Kramer MH, Kruip MJ, Kwakkel-van Erp JM, Leebeek FW (2006). Effectiveness of managing suspected pulmonary embolism using an algorithm combining clinical probability, D-dimer testing, and computed tomography. JAMA.

[67] Wells PS, Anderson DR, Bormanis J, Guy F, Mitchell M, Gray L, Clement C, Robinson KS, Lewandowski B (1997). Value of assessment of pretest probability of deep-vein thrombosis in clinical management. Lancet.

[68] Matsuo H, Nakajima Y, Ogawa T, Mo M, Tazaki J, Doi T, Yamada N, Suzuki T, Nakajima H: Evaluation of D-dimer in screening deep vein thrombosis in hospitalized Japanese patients with acute medical diseases/episodes. *Ann Vasc Dis* 2016, 2016 July. [Epub ahead of print]. 10.3400/avd.oa.16-00034 PMC5027256 27738461

[69] Park R, Seo YI, Yoon SG, Choi TY, Shin JW, Uh ST, Kim YK (2008). Utility of D-dimer assay for diagnosing pulmonary embolism: single institute study. Korean J Lab Med.

[70] Chen CJ, Wang CJ, Huang CC (2008). The value of D-dimer in the detection of early deep-vein thrombosis after total knee arthroplasty in Asian patients: a cohort study. Thromb J.

[71] Prisco D, Grifoni E (2009). The role of D-dimer testing in patients with suspected venous thromboembolism. Semin Thromb Hemost.

[72] Wang KL, Chu PH, Lee CH, Pai PY, Lin PY, Shyu KG, Chang WT, Chiu KM, Huang CL, Lee CY (2016). Management of Venous Thromboembolisms: part I. The consensus for deep vein thrombosis. Acta Cardiol Sin.

[73] Yamada N, Hirayama A, Maeda H, Sakagami S, Shikata H, Prins MH, Lensing AW, Kato M, Onuma J, Miyamoto Y (2015). Oral rivaroxaban for Japanese patients with symptomatic venous thromboembolism - the J-EINSTEIN DVT and PE program. Thromb J.

[74] Anderson DR, Kahn SR, Rodger MA, Kovacs MJ, Morris T, Hirsch A, Lang E, Stiell I, Kovacs G, Dreyer J (2007). Computed tomographic pulmonary angiography vs ventilation-perfusion lung scanning in patients with suspected pulmonary embolism: a randomized controlled trial. JAMA.

[75] Goodacre S, Sampson F, Thomas S, van Beek E, Sutton A (2005). Systematic review and meta-analysis of the diagnostic accuracy of ultrasonography for deep vein thrombosis. BMC Med Imaging.

[76] Stein PD, Fowler SE, Goodman LR, Gottschalk A, Hales CA, Hull RD, Leeper KV, Popovich J, Quinn DA, Sos TA (2006). Multidetector computed tomography for acute pulmonary embolism. N Engl J Med.

[77] Liew NC, Chang YH, Choi G, Chu PH, Gao X, Gibbs H, Ho CO, Ibrahim H, Kim TK, Kritpracha B (2012). Asian venous thromboembolism guidelines: prevention of venous thromboembolism. Int Angiol.

[78] Aujesky D, Obrosky DS, Stone RA, Auble TE, Perrier A, Cornuz J, Roy PM, Fine MJ (2005). Derivation and validation of a prognostic model for pulmonary embolism. Am J Respir Crit Care Med.

[79] Jimenez D, Aujesky D, Moores L, Gomez V, Lobo JL, Uresandi F, Otero R, Monreal M, Muriel A, Yusen RD, Investigators R (2010). Simplification of the pulmonary embolism severity index for prognostication in patients with acute symptomatic pulmonary embolism. Arch Intern Med.

[80] Zondag W, den Exter PL, Crobach MJ, Dolsma A, Donker ML, Eijsvogel M, Faber LM, Hofstee HM, Kaasjager KA, Kruip MJ (2013). Comparison of two methods for selection of out of hospital treatment in patients with acute pulmonary embolism. Thromb Haemost.

[81] Zondag W, Vingerhoets LM, Durian MF, Dolsma A, Faber LM, Hiddinga BI, Hofstee HM, Hoogerbrugge AD, Hovens MM, Labots G (2013). Hestia criteria can safely select patients with pulmonary embolism for outpatient treatment irrespective of right ventricular function. J Thromb Haemost.

[82] Landefeld CS, Goldman L (1989). Major bleeding in outpatients treated with warfarin: incidence and prediction by factors known at the start of outpatient therapy. Am J Med.

[83] Kuijer PM, Hutten BA, Prins MH, Buller HR (1999). Prediction of the risk of bleeding during anticoagulant treatment for venous thromboembolism. Arch Intern Med.

[84] Ruiz-Gimenez N, Suarez C, Gonzalez R, Nieto JA, Todoli JA, Samperiz AL, Monreal M (2008). Predictive variables for major bleeding events in patients presenting with documented acute venous thromboembolism. Findings from the RIETE registry. Thromb Haemost.

[85] Kearon C, Ginsberg JS, Kovacs MJ, Anderson DR, Wells P, Julian JA, MacKinnon B, Weitz JI, Crowther MA, Dolan S (2003). Comparison of low-intensity warfarin therapy with conventional-intensity warfarin therapy for long-term prevention of recurrent venous thromboembolism. N Engl J Med.

[86] Klok FA, Hosel V, Clemens A, Yollo WD, Tilke C, Schulman S, Lankeit M, Konstantinides SV (2016). Prediction of bleeding events in patients with venous thromboembolism on stable anticoagulation treatment. Eur Respir J.

[87] Klok FA, Barco S, Konstantinides SV (2017). External validation of the VTE-BLEED score for predicting major bleeding in stable anticoagulated patients with venous thromboembolism. Thromb Haemost.

[88] Cohen AT (2010). Asia-Pacific thrombosis advisory B: Asia-Pacific thrombosis advisory board consensus paper on prevention of venous thromboembolism after major orthopaedic surgery. Thromb Haemost.

[89] Bang SM, Jang MJ, Kim KH, Yhim HY, Kim YK, Nam SH, Hwang HG, Bae SH, Kim SH, Mun YC (2014). Prevention of venous thromboembolism, 2nd edition: Korean Society of Thrombosis and Hemostasis Evidence-Based Clinical Practice Guidelines. J Korean Med Sci.

[90] Fuji T, Fuijita S, Ujihira T, Sato T (2010). Dabigatran etexilate prevents venous thromboembolism after total knee arthroplasty in Japanese patients with a safety profile comparable to placebo. J Arthroplast.

[91] Fuji T, Fujita S, Kawai Y, Nakamura M, Kimura T, Fukuzawa M, Abe K, Tachibana S (2015). Efficacy and safety of edoxaban versus enoxaparin for the prevention of venous thromboembolism following total hip arthroplasty: STARS J-V. Thromb J.

[92] Fuji T, Fujita S, Kawai Y, Nakamura M, Kimura T, Kiuchi Y, Abe K, Tachibana S (2014). Safety and efficacy of edoxaban in patients undergoing hip fracture surgery. Thromb Res.

[93] Fuji T, Wang CJ, Fujita S, Kawai Y, Nakamura M, Kimura T, Ibusuki K, Ushida H, Abe K, Tachibana S (2014). Safety and efficacy of edoxaban, an oral factor Xa inhibitor, versus enoxaparin for thromboprophylaxis after total knee arthroplasty: the STARS E-3 trial. Thromb Res.

[94] Lee YJ (2014). Use of novel oral anticoagulants for the treatment of venous thromboembolism and its considerations in Asian patients. Ther Clin Risk Manag.

[95] Kuroda Y, Hirayama C, Hotoda H, Nishikawa Y, Nishiwaki A (2013). Postmarketing safety experience with edoxaban in Japan for thromboprophylaxis following major orthopedic surgery. Vasc Health Risk Manag.

[96] Kearon C, Akl EA, Ornelas J, Blaivas A, Jimenez D, Bounameaux H, Huisman M, King CS, Morris TA, Sood N (2016). Antithrombotic therapy for VTE disease: CHEST guideline and expert panel report. Chest.

[97] Cohen A, Chiu KM, Park K, Jeyaindran S, Tambunan KL, Ward C, Wong R, Yoon SS (2012). Managing venous thromboembolism in Asia: winds of change in the era of new oral anticoagulants. Thromb Res.

[98] Nakamura M, Yamada N, Ito M (2015). Current management of venous thromboembolism in Japan: current epidemiology and advances in anticoagulant therapy. J Cardiol.

[99] Wong WH, Yip CY, Sum CL, Tan CW, Lee LH, Yap ES, Kuperan P, Ting WC, Ng HJ (2016). A practical guide to ordering and interpreting coagulation tests for patients on direct oral anticoagulants in Singapore. Ann Acad Med Singap.

[100] Pradaxa® (dabigatran etexilate) now approved in more than 100 countries for stroke prevention in atrial fibrillation. Available at: https://www.boehringer-ingelheim.com/press-release/pradaxa-dabigatran-etexilate-now-approved-more-100-countries-stroke-prevention-atrial, March 6, 2014. Accessed October 19, 2017.

[101] Garcia DA, Baglin TP, Weitz JI, Samama MM, American College of Chest P (2012). Parenteral anticoagulants: antithrombotic therapy and prevention of thrombosis, 9th ed: American college of chest physicians evidence-based clinical practice guidelines. Chest.

[102] Consultation WHOE (2004). Appropriate body-mass index for Asian populations and its implications for policy and intervention strategies. Lancet.

[103] Kearon C, Ginsberg JS, Julian JA, Douketis J, Solymoss S, Ockelford P, Jackson S, Turpie AG, MacKinnon B, Hirsh J (2006). Comparison of fixed-dose weight-adjusted unfractionated heparin and low-molecular-weight heparin for acute treatment of venous thromboembolism. JAMA.

[104] Haas S (2008). New oral Xa and IIa inhibitors: updates on clinical trial results. J Thromb Thrombolysis.

[105] Samama MM, Poller L (1995). Contemporary laboratory monitoring of low molecular weight heparins. Clin Lab Med.

[106] Abbate R, Gori AM, Farsi A, Attanasio M, Pepe G (1998). Monitoring of low-molecular-weight heparins in cardiovascular disease. Am J Cardiol.

[107] Nieuwenhuis HK, Albada J, Banga JD, Sixma JJ (1991). Identification of risk factors for bleeding during treatment of acute venous thromboembolism with heparin or low molecular weight heparin. Blood.

[108] Bauer KA (2013). Pros and cons of new oral anticoagulants. Hematology Am Soc Hematol Educ Program.

[109] Chiang CE, Wang KL, Lip GY (2014). Stroke prevention in atrial fibrillation: an Asian perspective. Thromb Haemost.

[110] Nakamura M, Wang YQ, Wang C, Oh D, Yin WH, Kimura T, Miyazaki K, Abe K, Mercuri M, Lee LH (2015). Efficacy and safety of edoxaban for treatment of venous thromboembolism: a subanalysis of east Asian patients in the Hokusai-VTE trial. J Thromb Haemost.

[111] Wang Y, Wang C, Chen Z, Zhang J, Liu Z, Jin B, Ying K, Liu C, Shao Y, Jing Z (2013). Rivaroxaban for the treatment of symptomatic deep-vein thrombosis and pulmonary embolism in Chinese patients: a subgroup analysis of the EINSTEIN DVT and PE studies. Thromb J.

[112] Nakamura M, Nishikawa M, Komuro I, Kitajima I, Uetsuka Y, Yamagami T, Minamiguchi H, Yoshimatsu R, Tanabe K, Matsuoka N (2015). Apixaban for the treatment of Japanese subjects with acute venous Thromboembolism (AMPLIFY-J study). Circ J.

[113] Ahrens I, Lip GY, Peter K (2010). New oral anticoagulant drugs in cardiovascular disease. Thromb Haemost.

[114] Schulman S, Kearon C, Kakkar AK, Mismetti P, Schellong S, Eriksson H, Baanstra D, Schnee J, Goldhaber SZ, Group R-CS (2009). Dabigatran versus warfarin in the treatment of acute venous thromboembolism. N Engl J Med.

[115] Schulman S, Kakkar AK, Goldhaber SZ, Schellong S, Eriksson H, Mismetti P, Christiansen AV, Friedman J, Le Maulf F, Peter N (2014). Treatment of acute venous thromboembolism with dabigatran or warfarin and pooled analysis. Circulation.

[116] Bauersachs R, Berkowitz SD, Brenner B, Buller HR, Decousus H, Gallus AS, Lensing AW, Misselwitz F, Prins MH, Raskob GE (2010). Oral rivaroxaban for symptomatic venous thromboembolism. N Engl J Med.

[117] Buller HR, Prins MH, Lensin AW, Decousus H, Jacobson BF, Minar E, Chlumsky J, Verhamme P, Wells P, Agnelli G (2012). Oral rivaroxaban for the treatment of symptomatic pulmonary embolism. N Engl J Med.

[118] Prins MH, Lensing AW, Bauersachs R, van Bellen B, Bounameaux H, Brighton TA, Cohen AT, Davidson BL, Decousus H, Raskob GE (2013). Oral rivaroxaban versus standard therapy for the treatment of symptomatic venous thromboembolism: a pooled analysis of the EINSTEIN-DVT and PE randomized studies. Thromb J.

[119] Agnelli G, Buller HR, Cohen A, Curto M, Gallus AS, Johnson M, Masiukiewicz U, Pak R, Thompson J, Raskob GE (2013). Oral apixaban for the treatment of acute venous thromboembolism. N Engl J Med.

[120] Buller HR, Decousus H, Grosso MA, Mercuri M, Middeldorp S, Prins MH, Raskob GE, Schellong SM, Schwocho L, Segers A (2013). Edoxaban versus warfarin for the treatment of symptomatic venous thromboembolism. N Engl J Med.

[121] van Es N, Coppens M, Schulman S, Middeldorp S, Buller HR (2014). Direct oral anticoagulants compared with vitamin K antagonists for acute venous thromboembolism: evidence from phase 3 trials. Blood.

[122] Weitz JI, Haas S, Ageno W, Angchaisuksiri P, Bounameaux H, Nielsen JD, Goldhaber SZ, Goto S, Kayani G, Mantovani L (2016). Global anticoagulant registry in the field - venous Thromboembolism (GARFIELD-VTE). Rationale and design. Thromb Haemost.

[123] Seligsohn U, Lubetsky A (2001). Genetic susceptibility to venous thrombosis. N Engl J Med.

